# Supplementary material for: Fosfomycin, Applying Known Methods and Remedies to A New Era
Source: Diseases. 2020 Aug 7;8(3):31. doi: 10.3390/diseases8030031 (PMC7564589; doi:10.3390/diseases8030031)
Supplement: Supplementary file 1 [file diseases-08-00031-s001.pdf]

Supplementary Table 1.

Sample type	Service	Microoganism	Carbapenemase detection						Fosfomycin susceptibility test			
			mCIM	eCIM	KPC	NDM	MEM (µg/mL)	IPM (µg/mL)	MIC (µg/mL)	Interp.	DD (mm)	Interp.
Blood	ICU	*Klebsiella pneumoniae*	+	-	+	-	16	8	32	S	18	S
Blood	ICU	*Klebsiella pneumoniae*	+	-	+	-	>16	>16	32	S	19	S
Blood	ICU	*Klebsiella pneumoniae*	+	-	+	-	>16	>16	<16	S	19	S
Blood	ICU	*Klebsiella pneumoniae*	+	-	+	-	>16	>16	256	R	14	I
Blood	ICU	*Klebsiella pneumoniae*	+	-	+	-	>16	>16	256	R	15	I
Blood	Cardiology	*Klebsiella pneumoniae*	+	-	+	-	>16	>16	<16	S	23	S
Blood	ICU	*Klebsiella pneumoniae*	+	-	+	-	>16	>16	<16	S	21	S
Blood	Neurology	*Klebsiella pneumoniae*	+	-	+	-	>16	8	<16	S	18	S
Blood	ICU	*Klebsiella pneumoniae*	+	-	+	-	>16	>16	64	S	16	S
Blood	Pediatry	*Klebsiella pneumoniae*	+	-	+	-	>16	>16	<16	S	21	S
Blood	Pediatry	*Klebsiella pneumoniae*	+	-	+	-	>16	>16	32	S	17	S
Blood	Emergency	*Klebsiella pneumoniae*	+	-	+	-	>16	8	32	S	19	S
Blood	Emergency	*Klebsiella pneumoniae*	+	-	+	-	>16	>16	<16	S	18	S
Blood	Neonatology	*Klebsiella pneumoniae*	+	-	+	-	>16	>16	32	S	19	S
Blood	Neonatology	*Klebsiella pneumoniae*	+	-	+	-	>16	8	<16	S	19	S
Blood	Neonatology	*Klebsiella pneumoniae*	+	-	+	-	>16	8	32	S	20	S
Blood	ICU	*Klebsiella pneumoniae*	+	-	+	-	>16	>16	<16	S	20	S
Blood	Neonatology	*Klebsiella pneumoniae*	+	-	+	-	8	>16	<16	S	20	S
Blood	ICU	*Klebsiella pneumoniae*	+	-	+	-	>16	>16	<16	S	20	S
Blood	Oncology	*Klebsiella pneumoniae*	+	-	+	-	>16	>16	64	S	17	S
Blood	Neonatology	*Klebsiella pneumoniae*	+	-	+	-	>16	8	64	S	18	S
Blood	ICU	*Klebsiella pneumoniae*	+	-	+	-	>16	>16	32	S	17	S
Blood	ICU	*Klebsiella pneumoniae*	+	-	+	-	>16	>16	32	S	20	S
Blood	ICU	*Klebsiella pneumoniae*	+	-	+	-	>16	>16	128	I	13	I
Blood	ICU	*Klebsiella pneumoniae*	+	-	+	-	>16	>16	32	S	19	S
Blood	Emergency	*Klebsiella pneumoniae*	+	-	+	-	>16	>16	32	S	19	S
Blood	ICU	*Klebsiella pneumoniae*	+	-	+	-	>16	8	> 256	R	6	R
Blood	ICU	*Klebsiella pneumoniae*	+	-	+	-	>16	>16	> 256	R	6	R

Blood	Oncology	*Klebsiella pneumoniae*	+	-	+	-	>16	8	<16	S	20	S
Blood	ICU	*Klebsiella pneumoniae*	+	-	+	-	>16	>16	32	S	21	S
Blood	ICU	*Klebsiella pneumoniae*	+	-	+	-	>16	>16	32	S	19	S
Blood	ICU	*Klebsiella pneumoniae*	+	-	+	-	>16	>16	64	S	19	S
Blood	Hematology	*Klebsiella pneumoniae*	+		+	-	>16	>16	<16	S	23	S
Urine	Surgery	*Klebsiella pneumoniae*	+	-	+	-	>16	8	<16	S	21	S
Blood	ICU	*Klebsiella pneumoniae*	+	-	+	-	>16	8	<16	S	21	S
Urine	Nephrology	*Klebsiella pneumoniae*	+	-	+	-	>16	>16	128	I	22	S
Skin and soft tissue	Neurology	*Klebsiella pneumoniae*	+	-	+	-	>16	8	<16	S	20	S
Urine	Emergency	*Klebsiella pneumoniae*	+	-	+	-	>16	>16	> 256	R	15	I
Blood	ICU	*Klebsiella pneumoniae*	+	-	+	-	>16	>16	> 256	R	10	R
Skin and soft tissue	Oncology	*Klebsiella pneumoniae*	+	-	+	-	>16	8	> 256	R	6	R
Respiratory	Emergency	*Klebsiella pneumoniae*	+	-	+	-	>16	>16	<16	S	22	S
Urine	Internal Medicine	*Klebsiella pneumoniae*	+	-	+	-	>16	>16	<16	S	22	S
Urine	Nephrology	*Klebsiella pneumoniae*	+	-	+	-	>16	>16	32	S	19	S
Respiratory	ICU	*Klebsiella pneumoniae*	+	-	+	-	>16	>16	32	S	20	S
Blood	Neumology	*Klebsiella pneumoniae*	+	-	+	-	>16	8	<16	S	22	S
Respiratory	Neumology	*Klebsiella pneumoniae*	+	-	+	-	>16	>16	> 256	R	6	R
Skin and soft tissue	Oncology	*Klebsiella pneumoniae*	+	-	+	-	>16	>16	> 256	R	6	R
Blood	ICU	*Klebsiella pneumoniae*	+	-	+	-	>16	>16	<16	S	22	S
Respiratory	ICU	*Klebsiella pneumoniae*	+	-	+	-	>16	8	32	S	19	S
Urine	Emergency	*Klebsiella pneumoniae*	+	-	+	-	>16	>16	<16	S	19	S
Urine	Traumatology	*Klebsiella pneumoniae*	+	-	+	-	>16	8	32	S	20	S
Urine	External consultation	*Klebsiella pneumoniae*	+	-	+	-	>16	>16	> 256	R	6	R
Skin and soft tissue	ICU	*Klebsiella pneumoniae*	+	-	+	-	>16	>16	64	S	16	S
Urine	Emergency	*Klebsiella pneumoniae*	+	-	+	-	>16	>16	<16	S	22	S
Respiratory	Oncology	*Klebsiella pneumoniae*	+	-	+	-	>16	>16	<16	S	21	S
Respiratory	Emergency	*Klebsiella pneumoniae*	+	-	+	-	>16	>16	> 256	R	6	R
Blood	Surgery	*Klebsiella pneumoniae*	+	-	+	-	>16	>16	<16	S	23	S
Urine	ICU	*Klebsiella pneumoniae*	+	-	+	-	>16	>16	<16	S	20	S
Respiratory	ICU	*Klebsiella pneumoniae*	+	-	+	-	>16	>16	<16	S	20	S
Urine	Cardiology	*Klebsiella pneumoniae*	+	-	+	-	>16	8	64	S	16	S

Urine	ICU	*Klebsiella pneumoniae*	+	-	+	-	>16	>16	<16	S	22	S
Respiratory	ICU	*Klebsiella pneumoniae*	+	-	+	-	>16	8	64	S	17	S
Skin and soft tissue	ICU	*Klebsiella pneumoniae*	+	-	+	-	>16	>16	64	S	16	S
Blood	Oncology	*Klebsiella pneumoniae*	+	-	+	-	>16	>16	<16	S	22	S
Respiratory	Internal Medicine	*Klebsiella pneumoniae*	+	-	+	-	>16	>16	32	S	19	S
Respiratory	Neurology	*Klebsiella pneumoniae*	+	-	+	-	>16	>16	32	S	21	S
Urine	Gastroenterology	*Klebsiella pneumoniae*	+	-	+	-	>16	8	<16	S	21	S
Skin and soft tissue	Emergency	*Klebsiella pneumoniae*	+	-	+	-	8	>16	<16	S	21	S
Respiratory	Surgery	*Klebsiella pneumoniae*	+	-	+	-	>16	>16	32	S	19	S
Skin and soft tissue	Neurology	*Klebsiella pneumoniae*	+	-	+	-	>16	>16	32	S	20	S
Skin and soft tissue	Traumatology	*Klebsiella pneumoniae*	+	-	+	-	>16	>16	<16	S	20	S
Respiratory	Nephrology	*Klebsiella pneumoniae*	+	-	+	-	>16	>16	32	S	19	S
Respiratory	Emergency	*Klebsiella pneumoniae*	+	-	+	-	>16	>16	32	S	20	S
Respiratory	Surgery	*Klebsiella pneumoniae*	+	-	+	-	>16	8	32	S	20	S
Urine	ICU	*Klebsiella pneumoniae*	+	-	+	-	>16	8	<16	S	22	S
Urine	External consultation	*Klebsiella pneumoniae*	+	-	+	-	>16	>16	32	S	22	S
Urine	External consultation	*Klebsiella pneumoniae*	+	-	+	-	>16	>16	<16	S	21	S
Urine	External consultation	*Klebsiella pneumoniae*	+	-	+	-	>16	>16	<16	S	22	S
Respiratory	ICU	*Klebsiella pneumoniae*	+	-	+	-	>16	>16	32	S	20	S
Respiratory	Surgery	*Klebsiella pneumoniae*	+	-	-	-	>16	>16	64	S	18	S
Blood	Emergency	*Klebsiella pneumoniae*	+	-	+	-	>16	8	<16	S	21	S
Respiratory	Neurology	*Klebsiella pneumoniae*	+	-	+	-	>16	>16	<16	S	23	S
Urine	Neurology	*Klebsiella pneumoniae*	+	-	+	-	>16	>16	<16	S	24	S
Urine	Gastroenterology	*Klebsiella pneumoniae*	+	-	+	-	>16	>16	<16	S	21	S
Blood	Emergency	*Klebsiella pneumoniae*	+	-	+	-	>16	>16	> 256	R	6	R
Skin and soft tissue	Neonatology	*Klebsiella pneumoniae*	+	-	+	-	>16	>16	64	S	23	S
Liquid	Internal Medicine	*Klebsiella pneumoniae*	+	-	+	-	>16	>16	32	S	20	S
LCR	Neurology	*Klebsiella pneumoniae*	+	-	+	-	>16	8	32	S	20	S
Blood	ICU	*Klebsiella pneumoniae*	+	-	+	-	>16	>16	<16	S	23	S
Skin and soft tissue	Cardiology	*Klebsiella pneumoniae*	+	-	+	-	>16	>16	> 256	R	6	R
Respiratory	Neumology	*Klebsiella pneumoniae*	+	-	+	-	>16	>16	> 256	R	6	R
Skin and soft tissue	ICU	*Klebsiella pneumoniae*	+	-	+	-	>16	>16	> 256	R	6	R

Blood	Hematology	*Klebsiella pneumoniae*	+	-	+	-	>16	>16	> 256	R	6	R
Skin and soft tissue	ICU	*Klebsiella pneumoniae*	+	-	+	-	>16	>16	> 256	R	6	R
Respiratory	Neurology	*Klebsiella pneumoniae*	+	-	+	-	>16	>16	<16	S	20	S
Urine	ICU	*Klebsiella pneumoniae*	+	-	+	-	>16	8	> 256	R	6	R
Blood	ICU	*Klebsiella pneumoniae*	+	-	+	-	>16	8	> 256	R	6	R
Skin and soft tissue	ICU	*Klebsiella pneumoniae*	+	-	+	-	>16	8	32	S	18	S
Skin and soft tissue	Emergency	*Klebsiella pneumoniae*	+	-	+	-	>16	>16	64	S	18	S
Skin and soft tissue	Emergency	*Klebsiella pneumoniae*	+	-	+	-	>16	>16	32	S	17	S
Skin and soft tissue	Traumatology	*Klebsiella pneumoniae*	+	-	+	-	>16	8	<16	S	22	S
Skin and soft tissue	Traumatology	*Klebsiella pneumoniae*	+	-	+	-	>16	>16	<16	S	21	S
Respiratory	ICU	*Klebsiella pneumoniae*	+	-	+	-	>16	8	32	S	18	S
Respiratory	ICU	*Klebsiella pneumoniae*	+	-	+	-	>16	>16	<16	S	21	S
Respiratory	ICU	*Klebsiella pneumoniae*	+	-	+	-	>16	>16	<16	S	22	S
Blood	Surgery	*Klebsiella pneumoniae*	+	-	+	-	>16	>16	> 256	R	10	R
Blood	Internal Medicine	*Klebsiella pneumoniae*	+	-	+	-	>16	>16	<16	S	22	S
Respiratory	Internal Medicine	*Klebsiella pneumoniae*	+	-	+	-	>16	>16	32	S	21	S
Urine	Neumology	*Klebsiella pneumoniae*	+	-	+	-	>16	8	32	S	19	S
Respiratory	Neurology	*Klebsiella pneumoniae*	+	-	+	-	>16	>16	32	S	21	S
LCR	Emergency	*Klebsiella pneumoniae*	+	-	+	-	>16	>16	32	S	21	S
Liquid	Cardiology	*Klebsiella pneumoniae*	+	-	+	-	>16	>16	<16	S	24	S
Respiratory	Internal Medicine	*Klebsiella pneumoniae*	+	-	+	-	>16	>16	64	S	23	S
Sputum	ICU	*Klebsiella pneumoniae*	+	-	+	-	>16	>16	> 256	R	6	R
Blood	Surgery	*Klebsiella pneumoniae*	+	-	+	-	>16	>16	> 256	R	6	R
Skin and soft tissue	ICU	*Klebsiella pneumoniae*	+	-	+	-	>16	>16	> 256	R	6	R
Skin and soft tissue	Traumatology	*Klebsiella pneumoniae*	+	-	+	-	>16	>16	64	S	19	S
Skin and soft tissue	Surgery	*Klebsiella pneumoniae*	+	-	+	-	>16	>16	<16	S	22	S
Respiratory	ICU	*Klebsiella pneumoniae*	+	-	+	-	>16	>16	32	S	22	S
Blood	ICU	*Klebsiella pneumoniae*	+	-	+	-	>16	8	32	S	21	S
Blood	Hematology	*Klebsiella pneumoniae*	+	-	+	-	>16	>16	64	S	18	S
Respiratory	Emergency	*Klebsiella pneumoniae*	+	-	+	-	>16	8	64	S	20	S
Urine	Nephrology	*Klebsiella pneumoniae*	+	-	+	-	>16	8	32	S	22	S
Blood	Cardiology	*Klebsiella pneumoniae*	+	-	+	-	>16	8	128	I	19	S

Urine	ICU	*Klebsiella pneumoniae*	+	-	+	-	>16	>16	> 256	R	6	R
Respiratory	Internal Medicine	*Klebsiella oxytoca*	+	-	+	-	>16	>16	<16	S	23	S
Respiratory	ICU	*Klebsiella oxytoca*	+	-	+	-	>16	8	<16	S	24	S
Feces	ICU	*Klebsiella oxytoca*	+	-	+	-	>16	>16	<16	S	27	S
Catheter	Nephrology	*Klebsiella oxytoca*	+	-	+	-	>16	>16	<16	S	30	S
Blood	Cardiology	*Klebsiella ozaenae*	+	-	+	-	>16	8	64	S	20	S
Blood	Cardiology	*Escherichia coli*	+	-	+	-	>16	8	<16	S	29	S
Urine	Cardiology	*Escherichia coli*	+	-	+	-	>16	>16	<16	S	28	S
Liquid	Burn Unit	*Escherichia coli*	+	-	+	-	>16	8	<16	S	28	S
Blood	Nephrology	*Escherichia coli*	+	+	-	+	>16	8	<16	S	29	S
Blood	ICU	*Serratia marcescens*	+	-	+	-	>16	>16	32	S	25	S
Respiratory	ICU	*Serratia marcescens*	+	-	+	-	>16	8	32	S	25	S
Liquid	Surgery	*Morganella morganii*	+	-	+	-	>16	8	128	I	16	S
Skin and soft tissue	ICU	*Morganella morganii*	+	-	+	-	>16	8	256	R	10	R
Urine	Nephrology	*Citrobacter freundii*	+	-	+	-	>16	>16	<16	S	36	S
Urine	Internal Medicine	*Citrobacter freundii*	+	-	+	-	>16	>16	<16	S	35	S
Urine	Pediatry	*Citrobacter freundii*	+	-	+	-	>16	>16	<16	S	30	S
Respiratory	Neurology	*Citrobacter freundii*	+	-	+	-	>16	>16	<16	S	29	S
Skin and soft tissue	Surgery	*Citrobacter freundii*	+	-	+	-	>16	>16	<16	S	33	S
Urine	Internal Medicine	*Citrobacter freundii*	+	+	-	+	>16	8	256	R	6	R
Blood	Emergency	*Enterobacter cloacae*	+	-	+	-	>16	>16	<16	S	32	S
Skin and soft tissue	ICU	*Enterobacter cloacae*	+	-	+	-	>16	>16	> 256	R	6	R
Respiratory	Neurology	*Enterobacter cloacae*	+	-	+	-	8	>16	<16	S	32	S
Respiratory	Internal Medicine	*Enterobacter aerogenes*	+	-	+	-	>16	8	<16	S	23	S
Skin and soft tissue	Surgery	*Enterobacter aerogenes*	+	-	+	-	>16	8	32	S	21	S
Skin and soft tissue	Pediatry	*Citrobacter youngae*	+	-	+	-	>16	8	<16	S	23	S
